# DNA methylation mediates genotype and smoking interaction in the development of anti-citrullinated peptide antibody-positive rheumatoid arthritis

**DOI:** 10.1186/s13075-017-1276-2

**Published:** 2017-03-29

**Authors:** Weida Meng, Zaihua Zhu, Xia Jiang, Chun Lai Too, Steffen Uebe, Maja Jagodic, Ingrid Kockum, Shahnaz Murad, Luigi Ferrucci, Lars Alfredsson, Hejian Zou, Lars Klareskog, Andrew P. Feinberg, Tomas J. Ekström, Leonid Padyukov, Yun Liu

**Affiliations:** 1 Department of Biochemistry and Molecular Biology, The Ministry of Education Key Laboratory of Metabolism and Molecular Medicine, School of Basic Medical Sciences, Fudan University, West Building 13, 130 Dong An Road, Shanghai, China; 2 State Key Laboratory of Medical Neurobiology, Fudan University, Shanghai, China; 3 Division of Rheumatology, Huashan Hospital, Fudan University, Shanghai, China; 4 Institute of Environmental Medicine, Karolinska Institutet, Stockholm, Sweden; 5 Institute for Medical Research, Jalan Pahang, 50588 Kuala Lumpur, Malaysia; 6 Rheumatology Unit, Department of Medicine, Center for Molecular Medicine, Karolinska Institutet and Karolinska University Hospital, Stockholm, Sweden; 7 Institute of Human Genetics, Friedrich-Alexander-Universität Erlangen-Nürnberg (FAU), Erlangen, Germany; 8 Department of Clinical Neuroscience, Center for Molecular Medicine, Karolinska Institutet, Stockholm, Sweden; 9 Instramural Research Program, National Institute on Aging, National Institutes of Health, Baltimore, MD USA; 10 Center for Occupational and Environmental Medicine, Stockholm County Council, Stockholm, Sweden; 11 Center for Epigenetics and Departments of Medicine, Johns Hopkins University School of Medicine, Baltimore, MD USA

**Keywords:** Rheumatoid arthritis, Smoking, Epidemiology

## Abstract

**Background:**

Multiple factors, including interactions between genetic and environmental risks, are important in susceptibility to rheumatoid arthritis (RA). However, the underlying mechanism is not fully understood. This study was undertaken to evaluate whether DNA methylation can mediate the interaction between genotype and smoking in the development of anti-citrullinated peptide antibody (ACPA)-positive RA.

**Methods:**

We investigated the gene-smoking interactions in DNA methylation using 393 individuals from the Epidemiological Investigation of Rheumatoid Arthritis (EIRA). The interaction between rs6933349 and smoking in the risk of developing ACPA-positive RA was further evaluated in a larger portion of the EIRA (1119 controls and 944 ACPA-positive patients with RA), and in the Malaysian Epidemiological Investigation of Rheumatoid Arthritis (MyEIRA) (1556 controls and 792 ACPA-positive patients with RA). Finally, mediation analysis was performed to investigate whether DNA methylation of cg21325723 mediates this gene-environment interaction on the risk of developing of ACPA-positive RA.

**Results:**

We identified and replicated one significant gene-environment interaction between rs6933349 and smoking in DNA methylation of cg21325723. This gene-smoking interaction is a novel interaction in the risk of developing ACPA-positive in both Caucasian (multiplicative *P* value = 0.056; additive *P* value = 0.016) and Asian populations (multiplicative *P* value = 0.035; additive *P* value = 0.00027), and it is mediated through DNA methylation of cg21325723.

**Conclusions:**

We showed that DNA methylation of cg21325723 can mediate the gene-environment interaction between rs6933349 and smoking, impacting the risk of developing ACPA-positive RA, thus being a potential regulator that integrates both internal genetic and external environmental risk factors.

**Electronic supplementary material:**

The online version of this article (doi:10.1186/s13075-017-1276-2) contains supplementary material, which is available to authorized users.

## Background

Rheumatoid arthritis (RA) is a chronic autoimmune disease that leads to inflammation of the joints and surrounding tissues. It can cause severe functional disabilities, pain, and other disorders, such as cardiovascular disease. RA is a complex inflammatory disease affecting up to 1% of the population. The fact that the concordance rate for RA in monozygotic twins is less than 20% suggests that environmental factors may be highly involved in the etiology of the disease. In recent decades, it has been shown that the two major subgroups of RA, anti-citrullinated peptide antibody (ACPA)-positive and ACPA-negative RA, have in part different etiology. One example of this is the shared epitope (SE) alleles of the human leukocyte antigen DR beta chain 1 (HLA-DRB1), which is a major risk factor for ACPA-positive RA, but not to the same extent for ACPA-negative RA. Environmental/lifestyle factors, such as smoking [1–3] and other noxious airway exposures [4, 5], have been shown to be risk factors for RA, mainly for the ACPA-positive subset of RA.

Smoking is a well-studied risk factor for RA and for the severity of RA [6–8]. There is a dose-dependent interaction between smoking and variations in the *HLA*-*DRB1* gene in the risk of developing ACPA-positive RA [9, 10]. One hypothesis proposed for the etiology of ACPA-positive RA is that the autoantibodies (ACPA) that are directed against citrullinated proteins in the joints originate from the mucosal tissues, e.g. the lungs, exposed to harmful inhaled toxicants such as smoking or silica dust. However, there remains a challenge to fully understand the molecular mechanism of the gene-environment interaction in the pathogenesis RA.

**Fig. 1:**
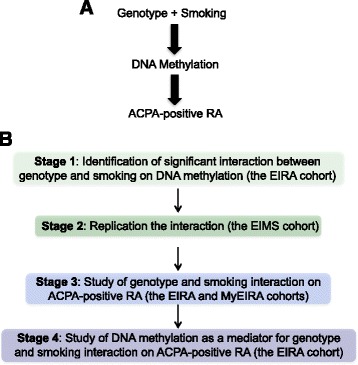
Study model (**a**) and work flow diagram (**b**). *ACPA* anti-citrullinated peptide antibodies, *RA* rheumatoid arthritis, *EIRA* Epidemiological Investigation of Rheumatoid Arthritis, *EIMS* Epidemiological Investigation of Multiple Sclerosis, *MyEIRA* Malaysian Epidemiological Investigation of Rheumatoid Arthritis

Epigenetic modifications, such as DNA methylation, have an important role in controlling when and where genes are expressed, and can be influenced by environmental factors. Such epigenetic modifications may thus provide a possible biological link between environmental exposures, genetic variations, and the disease. In fact, smoking has also been demonstrated to perturb DNA methylation signatures in lymphocytes [11]. Moreover, there is also growing evidence that epigenetic modifications can be controlled by the DNA sequence, and can be a mediator of genetic risk in common diseases, such as RA [12] and allergy [13]. Thus, it is relevant to investigate whether DNA methylation can mediate the interactions between genotype and smoking in the development of ACPA-positive RA (Fig. 1a) and whether it is a regulator that can integrate both internal genetic and external environmental risk factors.

In this report, by using data from multiple cohorts (Fig. 1b) we evaluated whether DNA methylation can mediate the interaction between genotype and smoking in the development of ACPA-positive RA.

## Methods

### Subjects

The EIRA (Epidemiological Investigation of Rheumatoid Arthritis) is a Swedish population-based case-control study. Recruitment of patients with RA in the EIRA study was described previously [14], and the healthy controls were selected from the same population to match the RA cases by age, sex and residential area at the time of diagnosis. Self-reported smoking habits were registered from the EIRA questionnaire. The genotyping and its quality control (QC) procedures have been described previously [14], and imputation was done using the IMPUTE2 algorithm [15] based on the phased 1000 genome reference set (March 2012 haplotypes). This group of samples with information on genotype, methylation, and smoking status was used for the investigation of genotype and smoking interaction in DNA methylation.

The EIMS (Epidemiological Investigation of risk factors for Multiple Sclerosis) is a population-based case-control study comprising Swedish-speaking subjects in Sweden and details of the recruitment procedure were described previously [16]. Briefly, newly diagnosed patients with multiple sclerosis (MS) were recruited via 40 study centers in Sweden and healthy controls were randomly selected from the national population register, matched by age, sex, and residential area. Self-reported smoking information was registered from the EIMS questionnaire.

The MyEIRA (Malaysian Epidemiological Investigation of Rheumatoid Arthritis) is another independent population-based case-control study, in which the subjects were recruited in Peninsular Malaysia with three major ethnic groups (i.e. Malays, Chinese, and Indians). The details of the MyEIRA study have been described elsewhere [3, 17]. In brief, patients with early RA were identified from nine rheumatology centers throughout Peninsular Malaysia, and for each case, a population control was randomly selected matched by age, sex, and residential area. All participants answered a questionnaire on a broad range of issues, including smoking habits.

The InCHIANTI study is a population-based prospective cohort study of residents from two areas in the Chianti region (Tuscany, Italy). The data collection started in September 1998 and was completed in March 2000 (baseline). A nine-year follow-up assessment of the InCHIANTI study population was performed in the year 2007–2008. Selection of participants and collection of DNA methylation data have been described previously [18, 19].

### DNA methylation measurement

Genome-wide methylation in peripheral blood cells from a subset of the EIRA, EIMS and InCHIANTI cohorts were evaluated by Illumina Infinium Human Methylation 450 BeadChip according to the manufacturer’s recommendations. Illumina Infinium Human Methylation 450 BeadChip array quantifies methylation levels at specific loci within the genome. The percentage methylation value for a particular CpG site, which represents the fraction of DNA with this CpG site methylated, was calculated on a scale of 0–1, per Illumina’s recommendations, using the formula:

$$ \mathrm{M}/\left(\mathrm{M} + \mathrm{U} + 100\right), $$

where M and U represent the methylated and unmethylated signal intensities, respectively. Methylation data from EIRA was published previously [12] and can be downloaded from Gene Expression Omnibus [GEO:GSE42861].

### Genotype and smoking interaction in DNA methylation analyzed using a linear regression model

We decided to focus on the single nucleotide polymorphisms (SNPs) within the major histocompatibility complex (MHC region) (chr6: 29,500,000–33,500,000 (hg19)) and the 10 differentially methylated positions (DMPs), which we identified previously to be associated with the development of ACPA-positive RA [12]. Details of methylation measurements have been provided previously [12]. Due to strong linkage disequilibrium of SNPs within the MHC region, we calculated the effective number of independent tests (*M*_*eff*_) by simpleM [20]. The total 1417 SNPs investigated in the study, which have minor allele frequency ≥0.05 and contain at least 10 individuals in each genotype group, represent 388 independent tests (*M*_*eff*_).

To identify genotype and smoking interaction in DNA methylation, we fit a linear regression model predicting methylation at each DMP as a function of genotype, smoking status (categorized as current smokers and never smokers) and their interaction term, and significant interaction was evaluated by calculating the interaction term in the model with a stringent Bonferroni-adjusted threshold of 0.05/(10 DMPs × 388 *M*_*eff*_) = 1.29 × 10^-5^. SNPs were treated with an additive minor-allele dosage model and potential confounding factors (that is, age, sex, and hybridization batch, and the first two principle components of cell-type proportions estimated by cell-specific methylation signatures as described before [12, 21]) were adjusted for in all analyses in both the EIRA and the EIMS cohorts.

### Genotype and smoking interaction in ACPA-positive RA

Two statistical models were used to evaluate the interaction between rs6933349 and smoking status (categorized as never smokers and current smokers, or as never smokers and ever smokers as will be specified later) in the development of ACPA-positive RA: (1) we performed interaction analysis by means of logistic regression with adjustment for age and sex, and interaction was evaluated on the multiplicative scale by calculating the interaction term in the logistic regression model, and (2) we also evaluated the interaction between genotype and smoking in ACPA-positive RA by departure from the additivity of effects, and biological interaction was estimated by calculating the attributable proportion due to interaction (AP). AP is the proportion of the incidence among individuals exposed to two interacting risk factors that is attributable to the interaction per se. AP >0 indicates that there is evidence for interaction on the additive scale. Confidence intervals for AP were estimated as described previously [22, 23]. Additionally, relative excess risk due to interaction (RERI) and the synergy index (SI) were also performed to evaluate interactions as described previously [24]. Both statistical models were also adjusted for ethnicity in analysis of the MyEIRA cohort.

### Linkage disequilibrium analysis

The measurement of linkage disequilibrium (*r* ^2^) between rs6933349 and the major RA *HLA-DRB1* SE alleles were calculated using genotype data from individuals in the EIRA cohort. The calculation was performed by using the “LD. Measures” function in the LDcorSV package.

### Mediation analysis

To investigate whether methylation of cg21325723 mediated the rs6933349 and smoking interaction in the risk of developing ACPA-positive RA, we assessed the effect of including DNA methylation as a covariate in the logistical regression models relating to rs6933349, smoking, and their interaction term to the ACPA-positive RA as the outcome. The 393 individuals from the discovery dataset analyzed, for whom we have complete information on genotype, smoking, methylation, and RA disease. Potential confounders (age and sex) were used for adjustment in all analyses.

## Results

### Stage 1: identification of an interaction between genotype and smoking in DNA methylation within the MHC region using a linear regression model

**Fig. 2:**
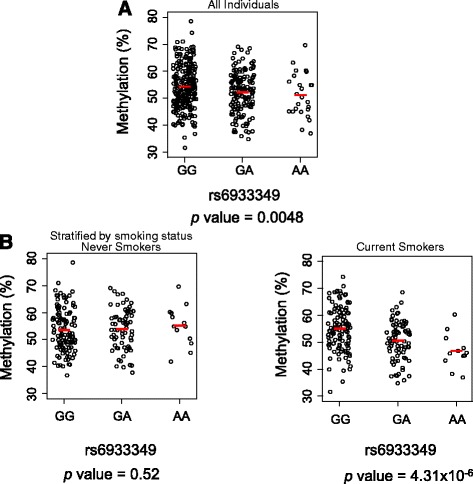
The associations between the genetic variant of rs6933349 and DNA methylation (cg21325723) in all individuals (**a**), and by smoking status (**b**), in the Epidemiological Investigation of Rheumatoid Arthritis (EIRA) study. Each *dot* represents an individual and *red horizontal bars* mark average DNA methylation levels. The statistical significance (*P* value) of association between genotype and DNA methylation, measured by linear regression model, is indicated at the *bottom* of the plot

As there are extremely large numbers of different combinations between SNPs and CpG sites, analyses of interactions, even in a relatively large cohort, are at risk of being underpowered. Thus, we decided to focus on the SNPs within the MHC region and the 10 DMPs, which we identified previously to be associated with the development of ACPA-positive RA [12]. By analyzing 393 samples from the EIRA cohort, for whom we have detailed information on genotype, DNA methylation in blood cells, and smoking status in each individual, we observed one significant interaction (Bonferroni-adjusted *P* value < 0.05) between a SNP, rs6933349 (chr6: 31002013), and smoking status in the DNA methylation level of the CpG site, cg21325723 (chr6: 32402555) (Additional file 1: Figure S1). We observed a significant association between DNA methylation and rs6933349 (*P* value = 0.0048) (Fig. 2a).

**Fig. 3:**
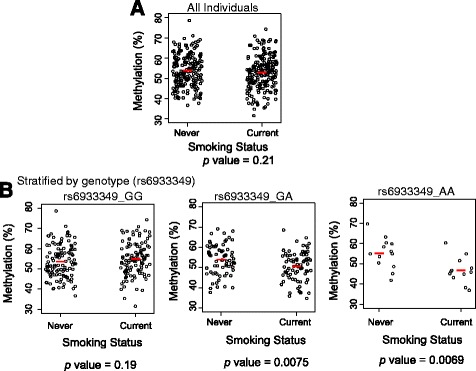
The associations between smoking and DNA methylation (cg21325723) in all individuals (**a**), and in individuals with rs6933349_GG, rs6933349_AG, or rs6933349_AA genotypes, respectively (**b**), in the Epidemiological Investigation of Rheumatoid Arthritis (EIRA) study. Each *dot* represents an individual and *red horizontal bars* mark average DNA methylation levels. The statistical significance (*P* value) of association between DNA methylation and smoking status, measured by Student’s *t* test, is indicated at the *bottom* of the plot

However, if we stratified the samples by smoking status, we observed significant association between the genotype and DNA methylation among current smokers (*P* value = 4.31 × 10^-6^), but not among never smokers (*P* value = 0.52) (Fig. 2b). Among current smokers, minor allele (rs6933349_A) carriers had a lower level of DNA methylation at cg21325723 (Fig. 2b), which was previously reported to be associated with increased risk of developing ACPA-positive RA [12] (*P* value = 1.49 × 10^-9^) (Additional file 1: Figure S2). Furthermore, among groups of individuals with a different genotype, we observed a different relationship between methylation level and smoking status (Fig. 3). For example, the DNA methylation level in current smokers who were carriers of rs6933349_GA or rs6933349_AA genotypes was significantly lower as compared to never smokers (*P* value = 0.0075 and 0.0069, respectively). No significant difference was seen in the DNA methylation level in current smokers and never smokers who were carriers of rs6933349_GG genotype (Fig. 3).

Next, we sought to confirm that the interaction between genetic variation and smoking in DNA methylation that was reported previously is not confounded by the RA status. We examined the interaction between rs6933349 and smoking in methylation of cg21325723 separately in healthy controls and ACPA-positive patients with RA, and observed consistent effects in both the control-only analysis (*P* value = 0.0037) and the patient-only analysis (*P* value = 0.019). In both analyses, we observed a significantly lower methylation level in rs6933349_AA genotype carriers among current smokers, while there were no associations between genotype and methylation among never smokers (Additional file 1: Figure S3). This showed that the combination of current smoking and minor allele (allele A) for rs6933349 is associated with hypo-methylation of cg21325723 and suggested the possibility of interaction between rs6933349 and smoking in the DNA methylation level of cg21325723, which was tested statistically and is described later within this paper.

Considering that DNA methylation is potentially dynamic, we further evaluated the stability of DNA methylation of cg21325723 in an independent dataset (the InCHIANTI cohort), in which methylation was measured in 460 individuals at two time points separated by 9 years. The Pearson’s correlation coefficient for cg21325723 was 0.722 (Additional file 1: Figure S4). This suggested that methylation in cg21325723 is moderately stable over 9 years. However, the level can still be altered, potentially by environmental risk factors for RA, such as smoking. This is consistent with a previous observation of familial clustering of changes in global DNA methylation over time [25].

### Stage 2: replication of an interaction between genotype and smoking in an independent data set

We continued to replicate the finding of interaction between rs6933349 and smoking in cg21325723 DNA methylation in an independent dataset, the EIMS, which is a population-based case-control study of MS. As the methylation level of cg21325723 was not associated with the MS status (*P* value = 0.71), we included a total of 139 healthy controls and 140 patients with MS, for whom information was available on genotype, methylation in blood, and smoking, for the replication study. In the combined meta-analysis using data from EIRA and EIMS, we observed significant interaction (*P* value = 2.18 × 10^-6^) between rs6933349 and smoking status (current vs. never smokers) in the methylation level of cg21325723. In the relatively small EIMS cohort alone, the interaction was marginally significant (*P* value = 0.08). Consistent with the finding from the EIRA, among the rs6933349_AA carriers from the EIMS cohort there was significantly lower methylation in current smokers (*P* value = 0.024), but not in never smokers (*P* value = 0.14) (Additional file 1: Figure S5).

### Stage 3: novel interaction between genotype and smoking impacting the risk of developing ACPA-positive RA

As hypo-methylation on cg21325723 has been associated with increased risk of developing ACPA-positive RA [12] (Additional file 1: Figure S2), we next investigated the interaction between rs6933349 and smoking in the risk of developing ACPA-positive RA. We addressed this question in a larger portion of the EIRA cohort (1119 healthy controls and 944 ACPA-positive patients with RA), which includes the subset of 393 individuals used in the discovery dataset. These analyses were performed separately for the models with multiplicative and additive effects.

#### Multiplicative model

This analysis of genotype and smoking interaction in ACPA-positive RA was evaluated on the multiplicative scale, which accesses the interaction term in the logistic regression model. We observed a marginally significant interaction (multiplicative *P* value = 0.022 in current smokers and 0.056 in ever smokers) between rs6933349 and smoking status in ACPA-positive RA (Table 1).

**Table 1:** The rs6933349 genotype and smoking interaction in the risk of developing ACPA-positive RA in the EIRA and MyEIRA studies

rs6933349	Smoking	OR (95% CI)	AP (95% CI)	Additive *P* value	Multiplicative *P* value
EIRA					
GG	Never	1.0 (ref)	0.315 (0.104–0.526)	0.00340	0.022
GG	Current	2.21 (1.74–2.82)			
GA/AA	Never	0.91 (0.72–1.15)			
GA/AA	Current	3.10 (2.38–4.05)			
EIRA					
GG	Never	1.0 (ref)	0.216 (0.04–-0.39)	0.01600	0.056
GG	Ever	1.68 (1.39–2.04)			
GA/AA	Never	0.91 (0.72–1.15)			
GA/AA	Ever	2.03 (1.66–2.49)			
MyEIRA					
GG	Never	1.0 (ref)	0.62 (0.29–0.96)	0.00027	0.035
GG	Ever	1.94 (1.30–2.90)			
GA/AA	Never	0.99 (0.78–1.26)			
GA/AA	Ever	5.14 (2.31–11.40)			

*AP* attributable portion, *EIRA* Epidemiological Investigation of Rheumatoid Arthritis, *MyEIRA* Malaysian Epidemiological Investigation of Rheumatoid Arthritis, *ref* reference

#### Additive model

**Fig. 4:**
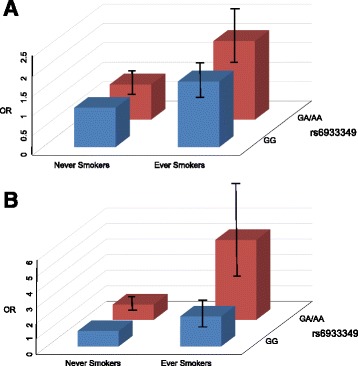
Odds ratios (*OR*) of developing anti-citrullinated peptide antibody-positive rheumatoid arthritis for different combinations of smoking and rs6933349 alleles in the Epidemiological Investigation of Rheumatoid Arthritis (*EIRA*) cohort (**a**) or the Malaysian Epidemiological Investigation of Rheumatoid Arthritis (*MyEIRA*) cohort (**b**)

This method is particularly relevant as it can further determine the degree of biological interaction between the two risk factors [26, 27]. In our analysis there was a statistically significant gene-environment interaction (additive *P* value = 0.0034 in current smokers and *P* value = 0.016 in ever smokers) between rs6933349 and smoking in the risk of developing ACPA-positive RA, with an attributable proportion due to the interaction (AP) value of 0.315 (95% CI 0.104 to 0.526) in current smokers and 0.216 (95% CI 0.040 to 0.392) in ever smokers (Table 1). We observed an increased risk of ACPA-positive RA in individuals who were ever smokers and carriers of rs6933349_AG/rs6933349_AA genotypes (OR = 2.03; 95% CI 1.66 to 2.49) (Table 1, Fig. 4a). In contrast, there was no interaction between the studied risk factors in relation to the risk of ACPA-negative RA (*P* value for AP = 0.74). Consistently, all other measures of interaction between rs6933349 and smoking in the risk of developing ACPA-positive RA were significant, with the RERI value of 0.978 (95% CI 0.178 to 1.778) in current smokers and 0.434 (95% CI 0.064 to 0.804) in ever smokers, and the SI value of 1.869 (95% CI 1.325 to 2.413) in current smokers and 1.729 (95% CI 1.114 to 2.344) in ever smokers.

This result was further replicated in the independent RA cohort, MyEIRA, a population-based case-control study performed in Malaysia [17]. We observed significant interaction (multiplicative *P* value = 0.035; additive *P* value = 0.00027) between rs6933349 and smoking (ever smokers) in the development of ACPA-positive RA (Table 1) in the MyEIRA cohort. Consistent with the finding from the EIRA, the combination of ever smoking and the genetic variant of rs6933349_A was associated with an increased risk of developing ACPA-positive RA (Fig. 4b), whereas no significant interaction was observed in the risk of ACPA-negative RA (additive *P* value = 0.45). The fact that the EIRA is mainly composed of Caucasians while the MyEIRA is a multiethnic population of Asian descent suggests that the interaction between rs6933349 and smoking in the risk of developing ACPA-positive RA is unlikely to be specific to certain ethnic groups.

### Independence from the shared epitope effect of rs6933349 in the interaction with smoking in the risk of developing ACPA-positive RA

The risk of developing ACPA-positive RA has been associated with interaction between smoking and *HLA-DRB1* SE alleles [17, 28]. Considering the complex structure of the MHC locus, we continued to investigate whether the newly identified SNP, rs6933349, which interacts with smoking to confer the risk of ACPA-positive RA, represents a novel interaction or simply reflects risk from *HLA-DRB1* SE alleles. We first examined the interaction between rs6933349 and smoking in the risk of developing ACPA-positive RA by means of logistic regression, with adjustment for *HLA-DRB1* SE alleles in the EIRA cohort. The multiplicative *P* value decreased from 0.05 to 0.018 after the SE adjustment, suggesting that this interaction is not dependent on *HLA-DRB1* SE alleles. Computation of the AP with adjustment for SE alleles in the EIRA cohort also points toward significant independent interaction between smoking and rs6933349 (AP = 0.32, *P* = 0.006).

Additionally, we investigated the linkage disequilibrium (LD) between rs6933349 and known *HLA-DRB1* SE alleles (including the major RA risk alleles HLA-DRB1*04:01 and *04:04), by calculating the *r* ^2^ value between them in the EIRA cohort. The SNP, rs6933349, which is in the region of MHC class I (Additional file 1: Figure S1), had no evidence of LD with *HLA-DRB1* SE alleles (*r* ^2^ ≤ 0.05) (Additional file 1: Table S1), suggesting that it represents a novel gene-environment interaction in the risk of developing ACPA-positive RA.

### Stage 4: DNA methylation as a potential mediator of the genotype and smoking interaction in the risk of developing ACPA-positive RA

Last, we explored the role of methylation of cg21325723 as a potential mediator of the interaction between rs6933349 and smoking in the risk of developing ACPA-positive RA (Fig. 1a). We performed mediation analysis [29] and modeled the relationships between rs6933349, smoking, cg21325723 methylation, and ACPA-positive RA in EIRA, using logistic regression. Using the multiplicative model, we observed significant interaction between rs6933349 and smoking in the risk of developing ACPA-positive RA (β coefficient = 0.99; 95% CI 0.30 to 1.68; *P* value = 0.0051). However, after including cg21325723 methylation as a covariate in the regression, the interaction between rs6933349 and smoking in relation to risk of ACPA-positive RA was attenuated and no longer significant (β coefficient = 0.39; 95% CI -0.39 to 1.17) (*P* value = 0.33). This result suggests that cg21325723 methylation may be a potential mediator of the gene-environment interaction between rs6933349 and smoking in the risk of developing of ACPA-positive RA.

## Discussion

In summary, we have identified a gene-environment interaction between rs6933349 and smoking on the DNA methylation level of cg21325723, which mediates the gene-environment interaction between rs6933349 and smoking in the risk of developing ACPA-positive RA. This gene-environment interaction represents a novel interaction in the risk of developing ACPA-positive RA in both Caucasian and Asian populations.

Information on smoking habits was gathered retrospectively by means of a questionnaire. The quality of exposure information may be different in cases and controls, which may result in recall bias. As misclassification of smoking is likely not related to genotype, the effects of such potential misclassification of exposure will be limited with regard to the investigated interaction between smoking and rs6933349.

An issue that may complicate studies involving epigenetics is that epigenetic modifications, such as DNA methylation, are much more dynamic than genetic variations and can be influenced by many confounders, such as age, sex, cell heterogeneity, and others. Although we addressed this issue by adjusting for these potential confounders in the linear regression model, there may still be other sources of confounding that were omitted and residual confounding that was not fully accommodated within the linear adjustment that we pursued. However, arguing against this idea is the fact that the identified gene-environment interaction between rs6933349 and smoking is not only important in the DNA methylation level of cg21325723, but is also important in the risk of developing ACPA-positive RA, and this was replicated in multiple ethnic groups.

With these limitations in mind, several reasonable inferences can however be made from these analyses. First, DNA methylation can act as a mediator of gene-environment interaction in the risk of developing ACPA-positive RA. To the best of our knowledge, this is the first report showing that DNA methylation can mediate gene-environment interaction regarding the development of a common disease. Even with the enormous success in genome-wide association studies (GWAS) of human common diseases in recent years, the search for new genetic risk factors has not revealed new strong effects [30]. It has been suggested that interactions between different genes and between genes and environment may explain a significant part of these risks. In this case, the associations between genetic variants and disease phenotype may be marginally significant and may have been neglected through a conventional genome-wide approach [31].

Even though multiple studies suggest that gene-environment interactions play an important role in disease susceptibility, including in RA [9, 10], a challenge remains to provide a functional interpretation and understand the molecular mechanism of the gene-environment interaction in the development of disease. DNA methylation, which can integrate both genetic and environmental cues, can be a possible “missing link” and is an attractive mechanism to explain the pathogenesis of disease. Combined with the fact that DNA methylation of cg21325723 can also integrate other RA genetic risk variants in the MHC class II locus [12], it is a possibility that DNA methylation is more proximal to the RA pathogenesis than genetic variations and this makes it a good candidate for therapeutic targets.

Second, even though we focused our study on the SNPs within the MHC region and selected CpG sites based on previous work, the success of identifying a CpG site, with methylation that mediates a novel interaction between genotype and smoking in the risk of developing ACPA-positive RA, suggests that this type of epigenetic regulation may be more common than currently acknowledged, and the findings reported here may simply be the “tip of the iceberg”. However, a challenge remains in genome-wide study of this issue, considering the extremely large number of combinations between SNPs and CpGs. Thus, further methodological work using advanced statistical methods for genome-wide evaluation, together with larger sample sizes and meta-analysis in independent studies, is important.

Third, the understanding of various possibilities of downstream epigenetic regulation explaining the detailed mechanisms awaits further study. Considering the critical role of DNA methylation in regulating gene expression, an attractive hypothesis is the epigenetic regulation of HLA gene expression, which in combination with particular *HLA-DR* gene products may be an important factor in exaggerated epitope presentation in RA. The altered DNA methylation profile within the MHC class II cluster in ACPA-positive RA, which may affect assembly, expression and peptide loading of HLA genes, would determine the functional capacity of RA-associated HLA class II molecules [32, 33] during presentation of autoantigen-derived peptides to T cells able to drive disease-inducing adaptive immunity (for further details of this scenario and its T and B cell specificities, see references [34–36]). In this scenario, it is important to identify a target HLA gene that has expression regulated by DNA methylation of cg21325723.

Finally, the approach presented here also demonstrates that it might be feasible to perform an integrated genetic and epigenetic analysis to identify genetic risk alleles for disease that are not found by conventional analysis. The association between the SNP, rs6933349, and methylation at cg21325723 was not genome-wide significant without considering the smoking interaction (Fig. 2a), and was neglected through a conventional genome-wide approach [12]. Given the data here, showing that part of genetic risk is mediated epigenetically, and also that epigenetic changes may integrate genetic and environmental effects, the augmentation of genetic studies with epigenetic analysis promises to illuminate hereditary risk that is otherwise opaque when considering genotype in isolation. Through identifying new risk factors and revealing the role of DNA methylation as mediator of genotype and smoking interaction in the development of ACPA-positive RA, this strategy will contribute towards understanding the mechanism of common disease.

## Conclusions

In conclusion, we identified one significant gene-environment interaction between rs6933349 and smoking in the DNA methylation levels of cg21325723, which has been shown previously to be associated with the risk of developing ACPA-positive RA. Additionally, we show that the gene-environment interaction between rs6933349 and smoking represents a novel interaction that confers the risk of developing ACPA-positive RA in different ethnic groups, and this gene-environment interaction is mediated through DNA methylation of cg21325723.

## Additional file

Additional file 1: Supplementary materials include five supplementary figures and a supplementary table. (PDF 440 kb)

## Abbreviations

ACPA: Anti-citrullinated peptide antibody
AP: Attributable proportion
DMP: Differentially methylated position
EIMS: Epidemiological Investigation of Multiple Sclerosis
EIRA: Epidemiological Investigation of Rheumatoid Arthritis
GWAS: genome-wide association studies
HLA-DRB: Human leukocyte antigen DR beta chain
InCHIANTI: Population-based prospective cohort study in two areas of Chianti
LD: linkage disequilibrium
*M*_*eff*_: Number of independent tests
MHC: Major histocompatibility complex
MS: multiple sclerosis
MyEIRA: Malaysian Epidemiological Investigation of Rheumatoid Arthritis
RA: Rheumatoid arthritis
RERI: Relative excess risk due to interaction
SE: Shared epitope
SI: Synergy index
SNP: Single nucleotide polymorphism
