# Supplementary material for: DNA methylation mediates genotype and smoking interaction in the development of anti-citrullinated peptide antibody-positive rheumatoid arthritis
Source: Arthritis Res Ther. 2017 Mar 29;19:71. doi: 10.1186/s13075-017-1276-2 (PMC5372280; doi:10.1186/s13075-017-1276-2)
Supplement: Supplementary file 1 — Supplementary materials include five supplementary figures and a supplementary table. (PDF 440 kb) [file 13075_2017_1276_MOESM1_ESM.pdf]

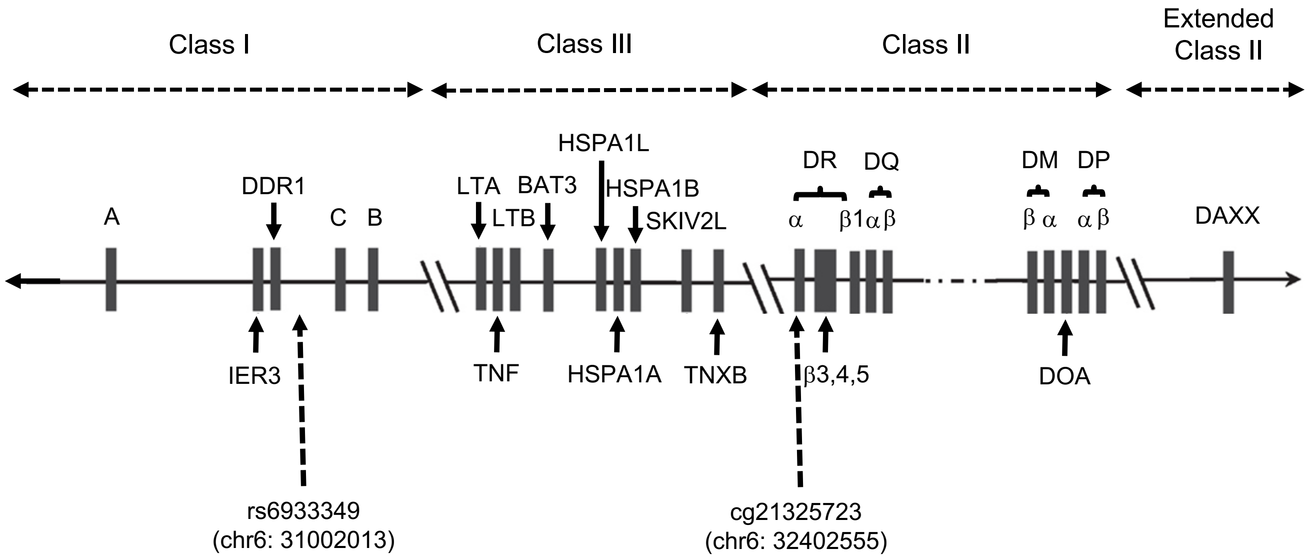

Supplementary figure S1 Schematic map of rs6933349 and cg21325723 within human major histocompatibility complex (MHC) region on the short arm of chromosome 6.

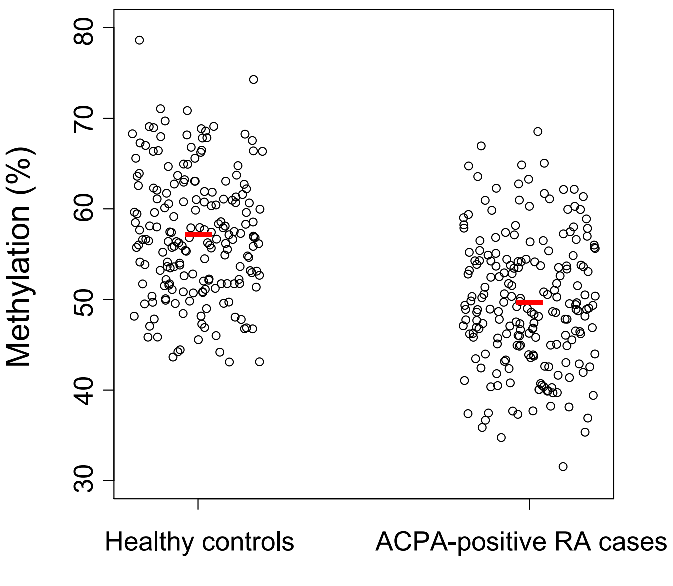

P value = 1.49E-9

Supplementary figure S2 The association between DNA methylation (cg21325723) and ACPA-positive RA in the EIRA study.

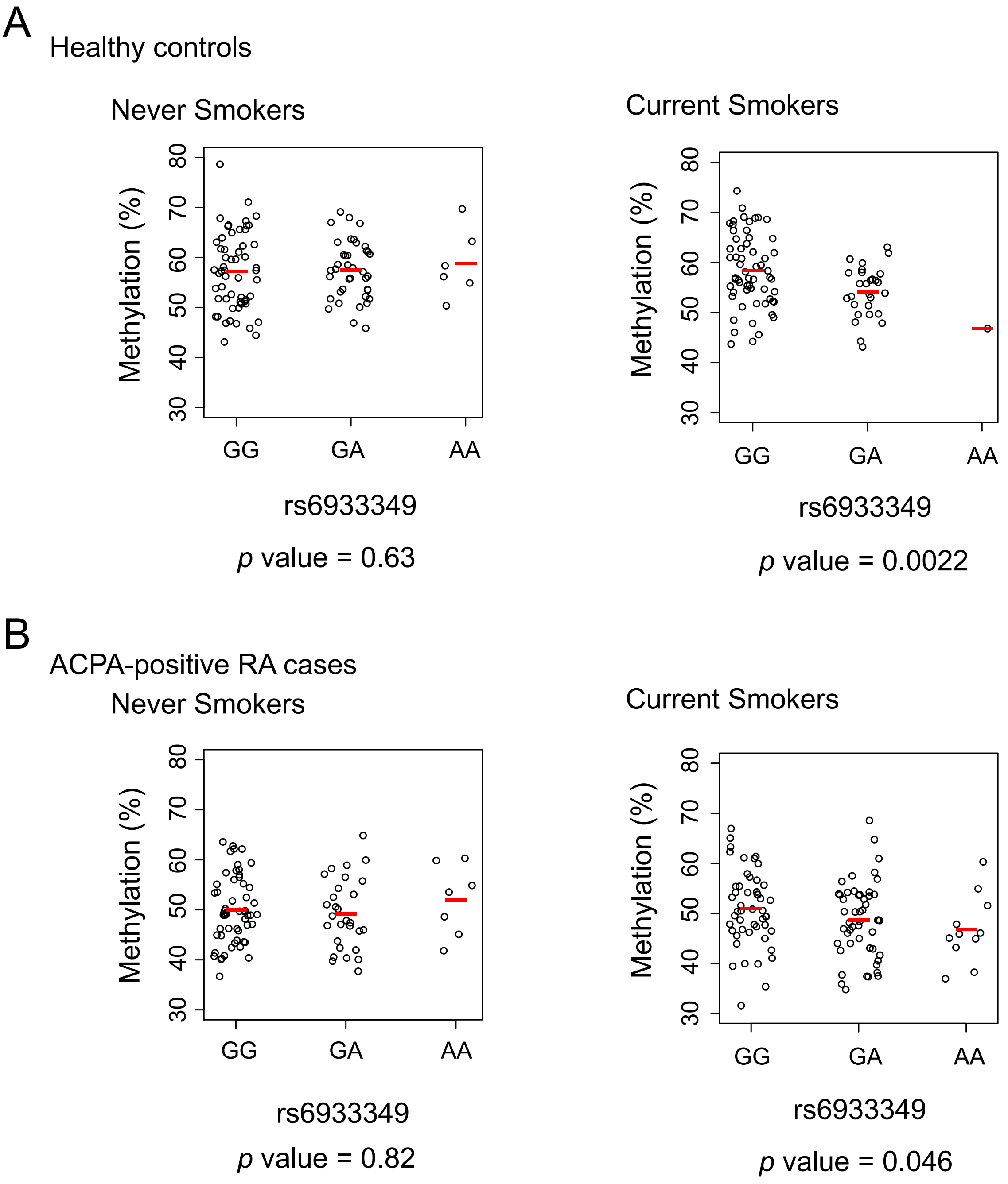

Supplementary figure S3 The associations between DNA methylation level on cg21325723 and genetic variant of rs6933349 in healthy controls (A), and in ACPA-positive RA cases (B), stratified by smoking status, in the EIRA study.

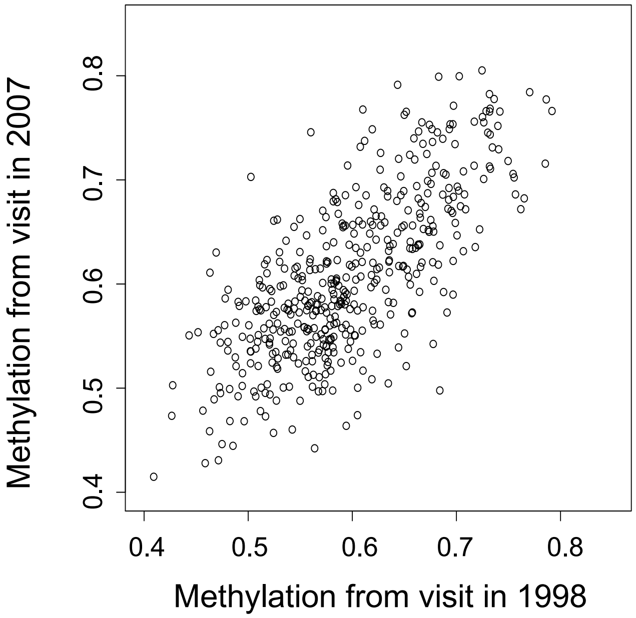

Pearson's correlation coefficient = 0.722

Supplementary figure S4 The correlation of cg21325723 methylation on the same individual separated by 9 years from the InCHIANTI cohort.

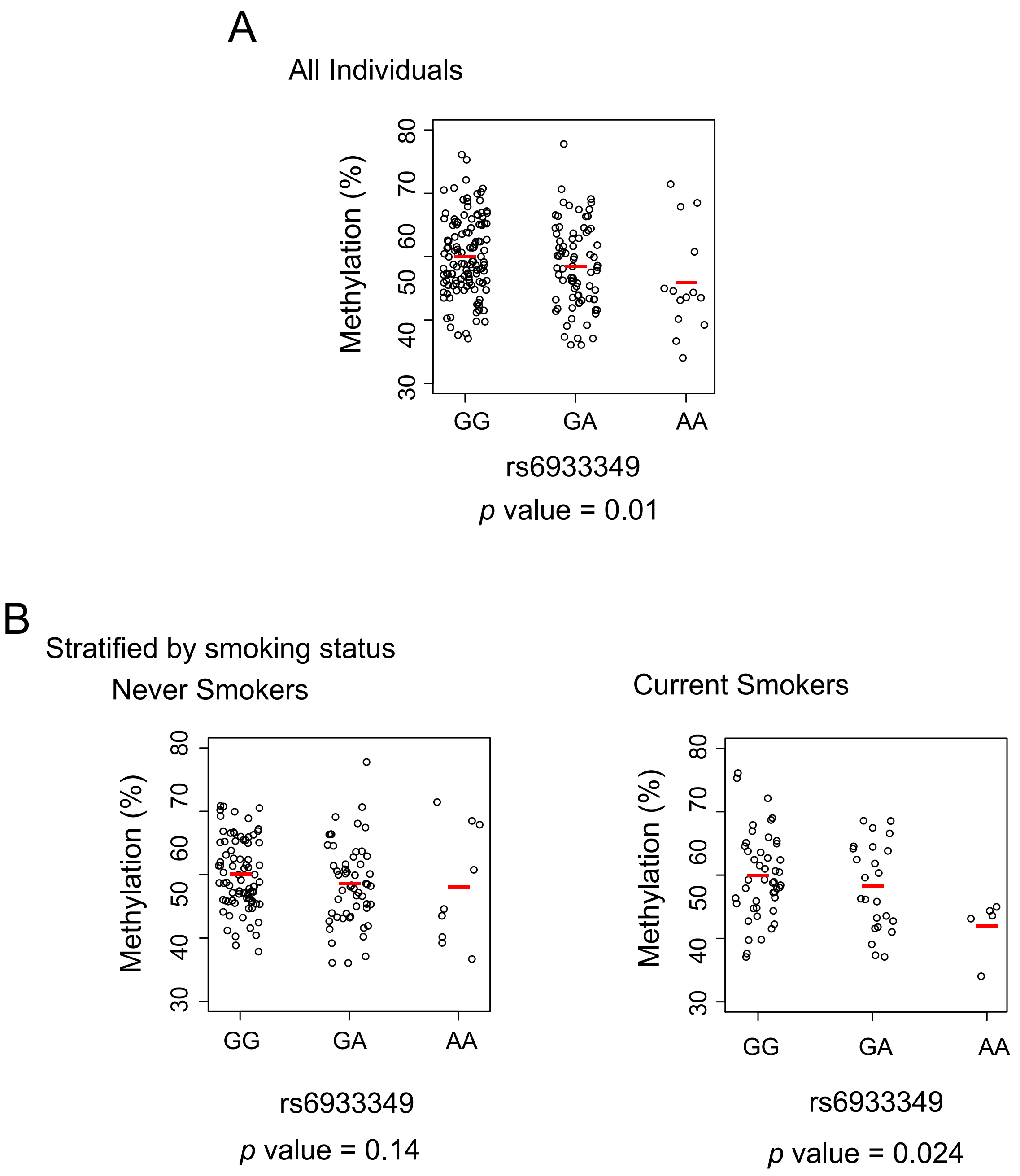

Supplementary figure S5 The associations between the DNA methylation level (cg21325723) and genetic variant of rs6933349 in all individuals (A), and in never smokers or current smokers in the EIMS study.

Supplementary table S1 Linkage disequilibrium (r^2) between the newly identified SNP, rs6933349, and the major RA risk shared epitope (SE) alleles

	SE	HLA-DRB1*04	HLA-DRB1*01	HLA-DRB1*10
rs6933349	1.11E-02	2.45E-02	8.57E-03	1.04E-03
